# Psychosocial family interventions for relatives of people living with psychotic disorders in the Arab world: systematic review

**DOI:** 10.1186/s12888-020-02816-5

**Published:** 2020-08-20

**Authors:** Aziza Al-Sawafi, Karina Lovell, Laoise Renwick, Nusrat Husain

**Affiliations:** 1 Division of Nursing Midwifery & Social Work, Faculty of Biology, Medicine and Health, The University of Manchester, College of Nursing/ Sultan Qaboos University of Manchester, Jean McFarlane Building Room 3.33 Oxford Road, Manchester, M13 9PL UK; 2 Mental Health in the Division of Nursing, Midwifery & Social Work, The University of Manchester, Oxford Road, Manchester, M13 9PL UK; 3 Division of Nursing, Midwifery and Social Work, The University of Manchester, Oxford Road, Manchester, M13 9PL UK; 4 Division of Psychology & Mental Health, The University of Manchester, Oxford Road, Manchester, M13 9PL UK

**Keywords:** *The* Arab world, Cultural adaptation, Family intervention, Schizophrenia, Psychotic disorders, Systematic review

## Abstract

**Background:**

Family interventions in schizophrenia are evidence based and have been adapted to different cultural settings to improve their effectiveness and acceptability. The Arab world has a unique set of socio-cultural norms and values that cannot be ignored when developing or implementing such interventions. There is a lack of research on the feasibility of delivering family interventions for schizophrenia in the Arab region. The aim of this review is to synthesise the available evidence about culturally-adapted psychosocial family interventions in the Arab world. The review identifies the content and characteristics of these interventions, determines the strategies used to adapt them to Arab culture successfully, assesses the feasibility and acceptability of the interventions, and evaluates the effectiveness of these interventions for service users and their families.

**Method:**

Five electronic databases were searched including MEDLINE, CINAHL, Cochrane Library, PsycINFO and EMBASE for articles written in Arabic and English from inception to August 2019. Data were extracted and synthesised narratively.

**Results:**

Six studies were retrieved from the search: three randomised control studies, two non-randomised studies and one qualitative study. There is limited evidence about culturally-adapted family interventions in the Arab region. However, the cultural adaptation process was comprehensive, and the implementation was reported to be feasible and acceptable. The methodological quality of the included studies was generally poor, so there is a risk of underestimating the effect size of the interventions due to lack of rigour and the presence of bias.

**Conclusion:**

The present review provides the foundation for future work regarding family interventions in the Arab world, and confirms the feasibility of implementing such interventions with some modifications. Furthermore, the data suggests that any family-oriented intervention for schizophrenia is likely to be better than standard care in improving the outcome for patients and their families.

## Background

Family interventions have been recognised as evidence-based practice and are recommended by national and international clinical guidelines [1–3]. Although there are multiple approaches for family interventions in psychotic disorders, the core components are problem-solving skills, psychoeducation, and communication skills [4, 5]. These interventions have multiple aims. First, they reduce any adverse effects from the family environment by building a good relationship with the family, educating the family, reducing over-involvement and critical comments, and changing any negative behaviours and beliefs that relatives might have [6]. Second, family interventions empower families with problem-solving and communication skills to enhance their capacity in handling stress and reducing the burden [7]. Third, interventions help the family anticipate likely problems and maintain realistic expectations regarding the patient [8].

Family interventions have consistently shown positive outcomes for individuals living with schizophrenia and their families [9–11]. However, a major criticism of such interventions is that they are based on Western models, and, therefore, may not apply to other countries without cultural adaptation [12]. Cultural adaptation is “the systematic modification of evidence-based treatment or intervention protocols to consider language, culture, and the context in such a way that is compatible with clients’ cultural patterns, meanings and values” [13]. Cultural adaptation aims to modify interventions to fit the cultural context of each diverse group to enhance acceptability, engagement, satisfaction and, ultimately, effectiveness [14–16]. A considerable amount of literature has suggested that cultural context influences all aspects of the diagnostic and treatment process [13, 17]. Therefore, people tend to accept and engage in interventions or treatment when these are congruent with their beliefs and values [14].

Recently, researchers have shown an increased interest in culturally adapting family interventions to different cultures to improve the acceptability and effectiveness of the treatment [12, 18–23]. These studies have shown that there are optimal benefits when interventions are tailored to a specific culture. A recent meta-analysis of culturally-adapted mental health interventions found a moderate to significant effect for such adaptations [14]. Multiple frameworks for cultural adaptation have been developed [15, 24, 25] to assist practitioners in providing culturally competent interventions when working with diverse clients. Bernal developed a three-stage process of adaptation when working with Hispanic populations, while others have focused on community-based formative approaches to therapy adaptation [25]. Furthermore, Bernal and Sáez-Santiago introduced linguistic translation to determine equivalence in addition to using a four-stage process of cultural adaptation which is consistent with Hawang and Berral (information gathering, preliminary adaptation, preliminary adaptation test, and adaptation refinement). However, none of these frameworks focus on family intervention for schizophrenia. Therefore, this study will follow the framework developed by Degnan et al. (2016) which includes nine themes: language, concepts and illness models, family, communication, content, cultural norms and practices, context and delivery, therapeutic alliance, and treatment goals [19]. The framework was developed based on a systematic review to analyse the nature and outcomes of culturally-adapted psychosocial interventions in schizophrenia. This comprehensive review, which included forty-six RCTs and 7828 participants, showed significant post-treatment effects in favour of adapted interventions. The review suggested a framework and concluded that the efficacy of the adapted intervention is proportional to the degree of cultural adaptation. In this review, the majority of studies were adapted for a majority population, which is unique compared to the other reviews, which were mainly for minority populations [14, 18, 26]. The heuristic model proposed by Degnan et al. (2016) provides clear guidance for cultural adaptation in comparison to previously conducted reviews. However, they included varieties of cultures and psychosocial interventions for schizophrenia. Furthermore, the available adaptation frameworks have mostly been developed in Western countries for minority groups, but might not work for indigenous populations like Arabs [14]. Therefore, our review will focus on culturally adapted family interventions in the Arab world.

The Arab region consists of 22 countries that share a common language, cultural traditions and history. Arab culture, including religion and tradition, plays a vital role in the political, social, and economic life of the region [27]. One important characteristic of Arab communities is the crucial role of the traditional family, which is considered the primary source of social support [28]. In Arab culture and tradition, the responsibility of caring for an ill family member is undertaken by the family itself [29]. This is also based on the interdependency within the Arab family unit, which outweighs the value of individual independence [29]. Therefore, seeking professional help is a family decision, and family members are the ones who demonstrate an interest in the wellbeing of the patient and help to carry out the treatment programme. Thus, it is very unusual for the patient to attend a psychiatric or medical practice alone. It is evident from the points mentioned above that the Arab family plays a decisive role in caring for the patient; therefore, this review will focus on family intervention for psychotic disorders.

In addition to Arab familial nuances, the Arab World has its own distinctive socio-cultural beliefs about mental illness, which heavily influence their help-seeking behaviours. First, Arabs associate the symptoms of mental illness with religious and cultural beliefs. They often believe that the cause of mental illness is due to evil spirits, demons, black magic or even as a result of God’s punishment [30]. As a response to such beliefs, traditional spiritual healers are sought out to treat such illnesses because they claim to be able to deal with the unknown [31]. In addition, due to the stigma of having a mental illness, many families opt to enlist the services of a spiritual healer. However, the use of these healers could hinder or delay actual preventative treatments. Second, Arabs usually somatise their psychological symptoms to avoid stigma [32]. The strong stigma attached to mental illness and the cultural belief in the evil eye (envy) prevent the family from disclosing various facts of family life to professionals [29]. Unfortunately, Arabs often have a negative view of people with mental illness, and the Arab cultural beliefs influence the definition, aetiology, clinical presentation, diagnosis and treatment of mental illnesses [33]. Therefore, knowledge of such factors is essential to provide culturally competent care and to avoid inappropriate delivery or poor engagement with family interventions [34].

There are many practical barriers to mental healthcare in Arab countries, such as literacy rates, lack of resources and trained healthcare providers [30]. In addition to scarce resources, the experience of mental illness is complicated by the disadvantages of war, poverty and stigma attached to mental illness. Consequently, culturally-adapted family interventions for psychotic disorders have the potential to improve the mental illness experience of Arabs globally. The intervention should be culturally relevant and delivered within the existing healthcare services to increase acceptability and ensure efficient use of available resources.

Despite the promising positive effect of family intervention in Western countries, this type of intervention has not yet been incorporated into the treatment for patients with psychotic disorders in the Arab world. To date, little is known about culturally-adapted family interventions for schizophrenia within Arab culture, making it challenging to design and test such interventions. In order to develop effective family interventions, the successes and failures of previous service-user and caregiver experiences require further exploration.

Finally, the purpose of this review, which is part of a larger study, is to synthesise the available evidence regarding culturally-adapted psychosocial family interventions in the Arab world. It will identify the content and characteristics of these interventions, determine the strategies used to successfully adapt these to Arab culture, assess the feasibility and acceptability of the interventions, and evaluate the effectiveness of culturally-adapted interventions for service users and their families.

## Methods

### Design

A mixed-method systematic review following the Preferred Reporting Items for Systematic Review and Meta-analysis was conducted [35]. The protocol is registered on PROSPERO with registration number: CRD42019117180 https://www.crd.york.ac.uk/prospero/

### Search strategy

Five electronic databases were searched including MEDLINE, CINAHL, Cochrane Library, PsycINFO and EMBASE for articles written in Arabic and English from inception to August 2019. The databases were searched using the keywords and their associated Medical Subject Heading (MESH) “schizophrenia or psychosis” AND “Arab or Bahrain or Egypt or Iraq or Jordan or Kuwait or Lebanon or Libya or Morocco or Oman or Palestine or Qatar or Saudi Arabia or Sudan or Syria or Tunisia or United Arab Emirates UAE or Yemen”. When the key terms of “family intervention or psychosocial intervention or psychoeducation” were added, it limited the number of results to only 5–8 studies. As there is no specific database that covers Arabic mental health journals, these were searched individually by finding the list of the journals from Google and by contacting researchers in the related field from different countries. Furthermore, reference lists of previous related systematic reviews and Arabic studies were hand searched to identify any additional relevant studies. An example of the search strategy for a PsycINFO is available in the supplementary material.

### Inclusion and exclusion criteria

Articles were included if they met the following inclusion criteria: 1) all study designs that evaluated or developed any type or format of culturally-adapted psychosocial family interventions in the Arab world. The interventions could be relevant psychoeducation, family therapy, counselling, communication and problem-solving skills training or CBT. 2) Participants who were relatives or family members caring for an individual with schizophrenia or related disorders. 3) The majority of carers (70% or above) were adults of 18 years or older, and the majority (70% or above) of people who had schizophrenia or related disorders based on ICD-10criteria. Articles were excluded if 1) The intervention did not include family members or caregivers 3) Languages were not Arabic or English.

### Screening

The results were exported to Covidence (www.covidence.org), an online software product that improves the efficiency of creating and maintaining systematic reviews. Based on predetermined inclusion and exclusion criteria, the team members independently undertook the initial screening of titles and abstracts. Two team members (A.S. and L.R.), independently screened the full texts of selected abstracts. A. S is a PhD student and L. R is a lecturer with a PhD. They both went through proper training and have experience in conducting and appraising systematic reviews. Areas of disagreement were resolved by consensus or by K. L, who is a professor and an expert in the field.

### Data extraction

The extraction sheet was developed in Excel and refined after piloting it on three articles. Data extraction elements included study details, intervention characteristics, adaptation process, feasibility and acceptability of the studies. The first author extracted the data from the articles and entered them into the data extraction form, and then another member of the team verified them. Team discussions resolved any discrepancies during the process of screening or extraction. The extraction sheet is available from the corresponding author.

### Methodological quality assessment

Given the methodological differences of the included studies, a range of quality appraisal tools were utilised. For RCTs, the Cochrane Collaboration’s tool for assessing the risk of bias was used [36]. It is a robust tool for assessing RCTs across six domains of risk (selection, performance, detection, attrition, reporting and other biases) [37]. For non-randomised studies, the tools used were adapted from JBI for non-randomised trials; and another JBI tool was used for qualitative studies [38]. These tools have been developed using a transparent process and have been tested in many previous systematic reviews (See additional file 1 for the tools).

### Data synthesis

Meta-analysis was not possible due to the diversity of designs, outcome measures and tools used. A narrative synthesis was conducted focusing on the objectives of the review to draw conclusions and generate areas for future work about family interventions in the Arab world [39, 40]. Quantitative and qualitative studies were analysed, and the results were narratively synthesised according to the framework proposed by Popay et al. [41]. The review included only one qualitative study; for this reason, it was reported narratively with the quantitative studies. The synthesis was initially conducted by the first author, and regularly discussed with the research team.

## Results

### Search results

**Fig. 1:**
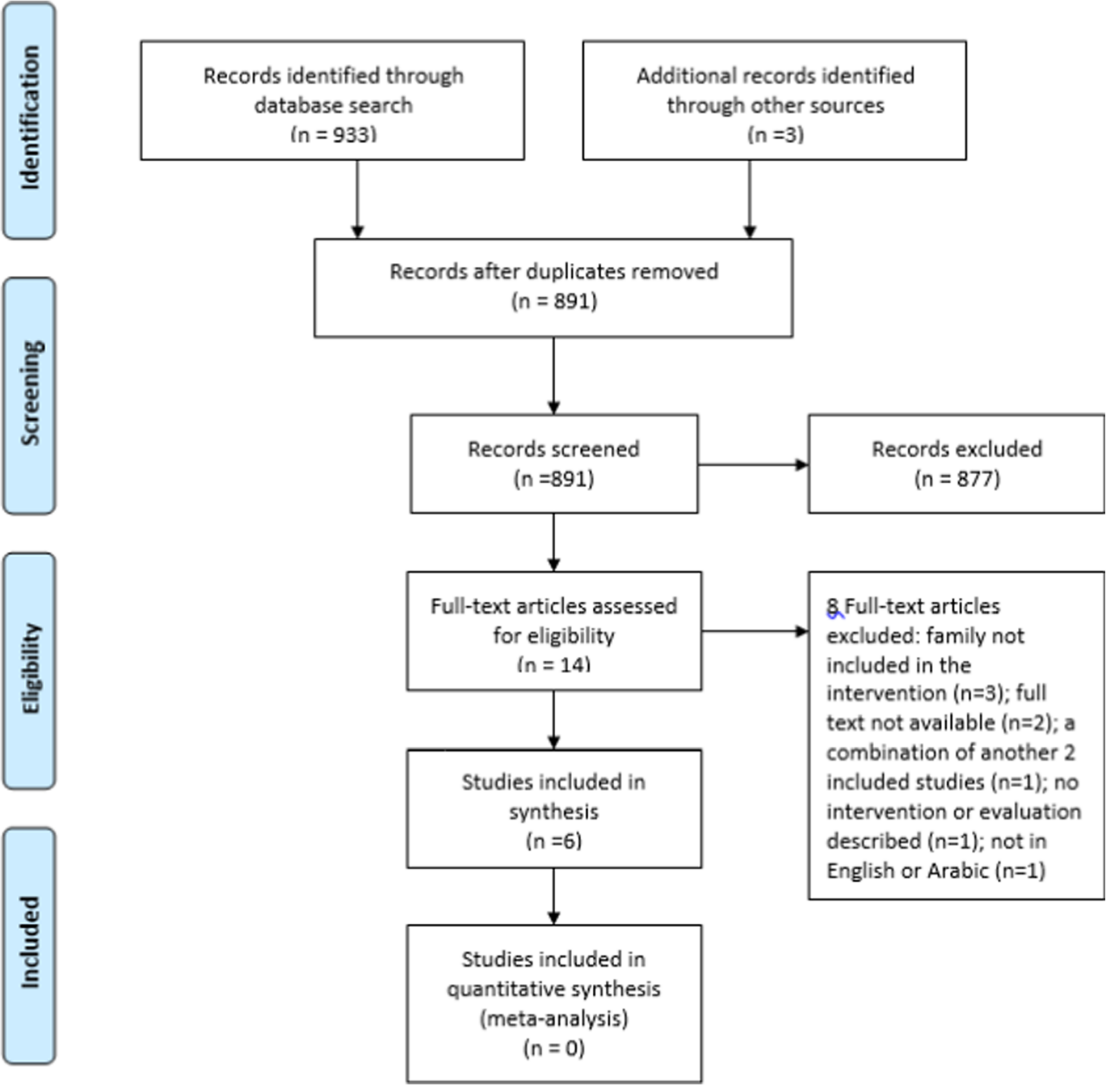
PRISMA (Preferred Reporting Items for Systematic Reviews and Meta-analyses) flow chart describing the study selection process, along with the reasons for exclusion

The database search yielded 933 titles and abstracts in addition to another three articles from hand searching. Following the removal of duplicates, 891 titles and abstracts were screened, after which 877 were excluded. The full-text articles of 14 references were obtained and considered against inclusion and exclusion criteria. Eight studies were excluded for different reasons, as shown in the PRISMA chart. This left six studies to be included in the final review (see Fig. 1 for the PRISMA flowchart).

### Study characteristics

Six studies met the inclusion criteria, including two studies from Jordan and four from Egypt. These were published between 2008 and 2018. In total, 394 patients and 344 caregivers were recruited into the studies. The designs were three RCTs [42–44], two non-randomised trials [45, 46], and one qualitative study [47]. See Table 1 for the descriptive characteristics of the studies.

**Table 1:** The Descriptive Characteristics of Studies

Study	Country	Aim	Sample size	Study design	Intervention/s	Main outcomes
Rami, et al. (2018) [42]	Egypt	to assess the effectiveness of patient and caregiver schizophrenia psycho-education program and its impact on improvement of psychopathology and quality of life (QoL)	Intervention: 30 patients with their caregivers Controls: 30 patients with their caregivers	Randomized, controlled, prospective intervention study	Culturally Sensitive Behavioral Family Psycho-Educational Program (BFPEP)	1- the rate of improvement of clinical variables including social functions 2- the adherence to medications 3- the quality of life of the patients
Hasan, et al. (2014) [43]	Jordan	to investigate the effectiveness of psychoeducational intervention delivered via a printed booklet on people diagnosed with schizophrenia and their primary caregivers’ outcomes	Interventions:58 dyads of patients and their caregivers Controls: 63 dyads of patients and their caregivers	a single blind RCT	Pycho-education by booklet	1- knowledge of schizophrenia 2- schizophrenia symptoms 3- Family Burden of Care 4- quality of life of caregivers
Al-Hadi Hsan, et al. (2017) [47]	Jordan	to explore potential processes underpinning any observed effect received from psychoeducational intervention via booklet in (Hasan et al., 2015)	8 patients and 9 caregivers	a qualitative process evaluation was undertaken, using audio-recorded face-to-face semi-structured interviews	Pycho-education by booklet	1- acceptance
Soliman, et al. (2018) [45]	Egypt	to assess the effectiveness of patient and caregiver schizophrenia psycho-education program and its impact on improvement of psychopathology and quality of life (QoL)	Intervention: 58 patients with their caregivers Controls: 58 patients with their caregivers	non- randomized control trial	Psycho-education	1- severity of symptoms 2- quality of life
El-Shafei, et al. (2008) [46]	Egypt	to establish a pilot study to examine the effect of family psycho-education and counselling on the outcome of schizophrenia especially regarding medication compliance, social functioning, clinical condition, relapse and hospitalizations	Interventions: 15 patients with their caregivers Controls: 15 patients with their caregivers	non- randomized control trial	Family psycho-education and counselling	1- the level of social functioning 2- medication compliance 3- clinical condition
Ahmed E & Ghaith H (2018) [44]	Egypt	to investigate the effect of psycho-educational program on perception of burden and attitudes toward mental illness among caregivers of patients with schizophrenia	Experimental group (25 caregivers) Control group (25 caregivers)	A quasi-experimental design	Psycho-education	1- caregiver burden 2- attitudes toward mental illness

### Quality assessment

As shown in Table 2, the methodological quality was good for Hasan et al. [43] and poor for the other randomized studies [42, 44]. Rami et al. [42] stated that randomisation was accomplished. However, they were not explicit about the method of randomisation or allocation concealment, which makes it open to selection bias. Additionally, the study protocol was not available to assess the reporting bias, and the study did not provide a hypothesis or power calculation. Multiple primary outcomes were evident, including clinical, social, quality of life and attitude towards medications. The primary outcome should be the one that has the existing evidence in direct association with the exposure of the intervention [48]. Accordingly, there should be one primary outcome in order to perform a power calculation. All statistical differences between arms were reported, but there was no report of an effort to minimise bias. The third study by Ahmed & Ghaith [44], was reported as a quasi-experimental design. Although they had a random assignment to the treatment and control group, the authors failed to provide a justification for the sample size of 50.

**Table 2:** RCT Quality Assessment

Article	Selection Bias		Performance Bias	Detection Bias	Attrition Bias	Reporting bias	Other Bias	Total score
	Random Sequence	Allocation Concealment	Blinding of Participants and Personnel	Blinding of Outcome Assessors	Incomplete Outcome Data	Selective Reporting	Anything Else	
Hasan et al. (2014) [43]	low	low	high	low	low	low	low	6/7
Rami et al. (2018) [42]	high	high	high	low	low	high	high	2/7
Ahmed E & Ghaith H (2018) [44]	Low	low	high	high	low	low	high	4/7

For non-randomised trials, the two studies had a high risk of bias because two or more criteria were not met according to the JBI tools [38] (see Additional file 2 for Methodological Quality of Non-randomised Trials). However, there is not enough data reported to judge the quality in many instances. The study, done by Soliman et al. [45], was reported as a cross-sectional interventional study. However, it may be more consistent with quasi-experimental design because of the lack of randomisation and inclusion of the control and intervention group. Furthermore, the dropout rate was not reported, which could have affected the analysis. The study by El-Shafei et al. [46], was reported as a case-control design, but the elements of the control group and randomisation make it more consistent with experimental studies. However, they failed to justify their sample size and did not include details of attrition, loss to follow-up or outcome measurements.

Overall, the quality of the included studies is poor, and none of these studies, except Hasan, et al. [43] had the statistical power to detect the benefit of family interventions. This indicates that the included studies have a risk of underestimating the effect size of interventions because of a great risk of type II error.

The qualitative study conducted by Al-HadiHsan et al. (2017) is consistent with good quality studies according to the JBI tool [38]. The study did not follow any methodological theory for qualitative research because the authors were trying to answer the research question and explain the quantitative data. Two questions in the appraisal tool were not reported. First, locating the researcher culturally or theoretically in the study. Second, the acknowledgement of the potential influence of the researcher in the study and vice versa. Although these two points were not reported, they are more applicable to different qualitative methodological theories that were not followed in this study.

### Intervention characteristics

Interventions in the six studies were delivered in Egypt [4] and Jordan [2]. The qualitative study [47] was the second stage of another study in the review [43]. Despite the differences in the content, all the studies shared the same component of psychoeducation, and two included communication and problem-solving skills [42, 43], while ElShafei et al. (2006) used counselling sessions. Furthermore, they varied in terms of intervention characteristics such as mode of delivery, duration and number of sessions. All studies except Ahmed & Ghaith [44] were individual-family sessions and were attended by patients and their caregivers. Four of the studies were delivered in a clinical setting in the outpatient department [42, 44–46], and one was delivered using a booklet inside the patients’ home [43]. The duration of the intervention ranged from 8 weeks to 6 months. The duration of an individual session was reported in two studies as 60 min [42, 44]. Healthcare providers or researchers led all the interventions; none of which were delivered in inpatient settings. All the studies compared family intervention to standard care. (See Table 3 for the Intervention Characteristics Table.

**Table 3:** Intervention Characteristics Table

author	Adaptation Model	Type of Intervention	Components of the Intervention	Model (Group/ Individual)	Setting	Intervention Attendees	No. of Session/Duration/ Frequency	Duration of Intervention	Delivery - Method	Therapist/ Training
Rami et al. (2018) [42]	BFT manual by (Mueser and Glynn, 1999). The educational component was adapted from the psychoeducational program by ElShafie and colleagues (2002).	Culturally sensitive Behavioral Family Psycho-Educational Program (BFPEP).	psycho-education + communication enhancement training + problem-solving skills training	Individual	Outpatient clinic	Caregivers and patients	14 one-hour sessions (weekly in the first 2 months, twice/month in the second 2 months, then every 3 weeks for the last 2 months)	Over 6 months	Individual family session in a bifocal format	Researchers/ no training reported
Hasan et al. (2014) [43]	Based on the framework of Atkinson and Coia [24]. it covers Bloom’s taxonomy of learning domains	Psycho-education by booklet	psycho-education + stress management strategies + problem-solving intervention	Individual	Community/ sent to patients’ home	Caregivers and patients	A psychoeducational booklets each fortnight	12 weeks	Booklet	Not applicable
Soliman et al. (2018) [45]	Not reported	Psycho-education	Mainly psycho-education	Individual	Outpatient during follow-up	Caregivers and patients	6 (one sessions/month)/ duration not reported	6 months	Psycho-education sessions during follow-up sessions	Psychiatrists
(El-Shafei et al., 2008) [46]	Not reported	psycho-education	Psycho-education + counselling sessions	Individual	Outpatient	Caregivers and patients	Not reported	Not reported	Brief education + counselling sessions	Researchers
Ahmed E & Ghaith H (2018) [44]	No adaptation	psycho-education	Psycho-education involving	Group	Outpatient	Caregivers	12 2 introductory and 10 interventional	2 months	Lecture, group discussion, question and answer methods, and demonstrations	Not reported

### Contents and components of the interventions

Two studies reported the process of adaptation and modification of the original manuals. Hasan et al. (2014) used the framework of Atkinson and Coia, which covers Bloom’s Taxonomy of Learning Domains, while Rami et al. [42] used the Behavioural Family Therapy (BFT) manual by Mueser and Glynn (1999). However, for the component of psychoeducation, they adopted the programme prepared by ElShafie and colleagues (2002), which was developed specifically for Egyptians.

First, the psychoeducation components included signs, symptoms, aetiology, diagnosis, treatment, as well as relapse signs and management strategies for schizophrenia. Additionally, it included facts and myths about schizophrenia, and how these effect the persons’ thoughts, emotions, and behaviour. The treatment component included information about medication, its side effects, anticipated benefits of the medicine, non-pharmacological treatment, adherence to treatment, the importance of follow-up, and information regarding prognosis. Furthermore, leaflets were distributed to participants during sessions that had information regarding schizophrenia, high expressed emotion families, notes and homework assignments for problem solving, and communication skills training [42]. Second, communication enhancement training included learning skills for active listening, delivering positive and negative feedback, and requesting changes in each other’s behaviours. Third, problem-solving skills training included identification of specific family problems and practical advice for solving them, such as using cognitive and behavioural techniques for managing a patient’s symptoms. Fourth, the stress vulnerability model addressed the role of the family, burden of care, and stress management skills and strategies.

### Strategies used to adapt the intervention

The strategies for adaptation included different themes, but the common themes in all studies were language, context and delivery, and family. Language adaptation was reported in all studies; the content was modified and translated into simple Arabic, and the complexity of psychoeducation was simplified. The context and delivery adaptation were reported in most studies, as researchers delivered the intervention in individual therapy sessions instead of groups to facilitate the cultural context of Arabs. All studies acknowledged the important role of the family and its distinct structure and processes.

Rami et al. (2018) was the only study which reported a detailed process of cultural adaptation. They piloted the intervention before the actual study to assess the acceptability and linguistic accessibility, and they modified the intervention accordingly. Moreover, the theme of concepts and illness models was incorporated by increasing the number of sessions regarding the biological basis of the illness from one session in the original BFT manual to two. They adapted the content to incorporate cultural norms and practices by including folk stories relevant to the cultural and religious beliefs of the participants. Further to these adaptations, the programme in Rami, et al. [42] was shortened to 6 months instead of nine because of practical and financial reasons that may have influenced adherence and attendance.

### Feasibility and acceptability of the interventions

Feasibility included the assessment of recruitment, attendance, retention (the proportion of participants who complete therapy sessions) and the compatibility of the interventions with available resources. All studies reported a feasible recruitment process without significant barriers or difficulties. Attendance was also feasible because three studies [42, 44, 45] delivered the interventions during follow-up appointments, which ensured a high level of attendance. The fourth study by Hasan et al. [43] was delivered via a booklet at patients’ homes. Two studies did not report attendance [44, 46]. The assessment of retention was reported in only two studies [42, 43]. Rami et al. [42] reported that [4] participants from the case group and [6] participants from the control group missed their regular sessions. The attrition rate in the Hasan et al. [43] study was [6] from the intervention group and [10] from the control group. All the studies reported compatibility of the intervention with available resources. The study by Rami et al. [42] reported that the intervention was applicable and accessible because of the brevity of the programme. Consequently, the feasibility of the programme was enhanced by meeting the needs of caregivers.

Acceptability is defined as “a multi-faceted construct that reflects the extent to which people delivering or receiving healthcare intervention consider it to be appropriate, based on anticipated or experienced cognitive and emotional responses to the intervention” [49]. Hasan et al. [43] followed his trial with a qualitative study to assess the acceptability of interventions. Qualitative interviews with service users and caregivers confirmed the acceptability of the interventions and found that the booklets were appropriate and valuable. No other studies examined acceptability.

### Effect of interventions

The outcomes reported across the studies varied, and most of them did not distinguish between primary and secondary outcomes. The most frequently reported outcome was severity of symptoms using the Positive and Negative Syndrome Scale (PANSS) [42, 45, 46]. The four studies reported a statistically significant difference between the two groups concerning positive and negative symptoms experienced by service users, which favoured the intervention group. Additionally, Hasan et al. [43] reported a reduction in the severity of symptoms at the three-month follow-up. Other frequently reported outcomes were social functioning, adherence to medication, quality of life and knowledge of schizophrenia. Two studies assessed family outcomes, which included the family burden of care, attitude and carers’ quality of life. (See Table 4 for the results of each outcome).

**Table 4:** the Results

Author	No. of Participants	Outcome (Primary or Secondary)	Scale	Result for Each Outcome
Eman S. Soliman	116 patients and their caregivers.	Severity of symptoms (did not specify)	Positive and Negative Syndrome Scale (PANSS)	There is a statistically significant difference between group A patients, who received PCSPP, and group B patients, who received TAU, as regards positive, negative, general psychopathology symptoms, and total scores, with a higher score toward TAU.
		Quality of life (did not specify)	World Health Organization Quality of Life Questionnaire-short version (WHOQoL-BREF) (Arabic version) M4	There is a statistically significant difference between group A and group B regarding question 1, question 2, domain 1 (physical), domain 2 (psychological), domain 3 (social relation), and domain 4 (environment) measured by WHOQoL scale, with a higher score in patients who received PCSPP.
Hisham Rami	60 patients and their caregivers	The rate of improvement of clinical variables (primary)	The Positive and Negative Syndrome Scale (PANSS)	A statistically significant difference (*p* < .05) between pre- and post-treatment scores in patients with schizophrenia in the case group receiving the BFPEP on the PANSS.
		Social functions (primary)	The Social Functioning Questionnaire (SFQ)	A statistically significant difference (p < .05) between pre- and post-treatment scores in patients with schizophrenia in the case group receiving the BFPEP on the SFQ and all of their subscales, indicating better social functioning at post-treatment.
		The adherence to medications (primary)	Drug Attitude Inventory (DAI) (Hogan, Awad & Eastwood, 1983)	Found a statistically significant difference (p < .05) between patients with schizophrenia in the case group receiving the BFPEP and patients with schizophrenia in the control group receiving STU regarding intervention outcome measures on the DAI10 indicating better drug attitude.
		Quality of life of the patients (primary)	Quality of Life scale (QLS) (Heinrichs, Hanlon & Carpenter, 1984)	Better quality of life at post-treatment in the intervention group receiving the BFPEP compared to STU.
El-Shafei	30 patients and their caregivers	Clinical condition (did not specify)	Positive And Negative Syndrome Scale (PANSS) (Kay, et al., 1987).	A significant improvement in the total PANSS occurred in patients in the intervention and not the control group over time.
		The level of social functioning (did not specify)	Social Functioning Questionnaire (SFQ) (Clifford 1987)	Statistically, significant improvement was detected in the social functioning of patients in the case group compared to controls over time both on Total SFQ.
		Medication compliance (did not specify)	The Drug Attitude Inventory (DAI) (Awad, 1993)	A significant improvement in compliance and attitude towards psychotropic medications when using these measures as compared to controls.
Abd Alhadi Hasan	112 dyads of patients and their caregivers	Knowledge of schizophrenia (primary)	Knowledge about Schizophrenia Questionnaire (KASQ)	Participants in the intervention group had statistically significant improvements in KASQ scores at post-treatment and three-month follow-up.
		schizophrenia symptoms (secondary)	Positive and Negative Syndrome Scale (PANSS) for PDWs	PANSS scores show that intervention was associated with a reduction in symptom severity at post-treatment and three-month follow-up.
		Family Burden of Care and quality of life (secondary)	Family Burden Interview Scale (FBIS) Schizophrenics’ Carers’ Quality of Life Scale (S-CQoL)	The group and time effect were statistically significant for all primary caregiver outcomes over different follow-up times.
Ahmed E & Ghaith H (2018)	50 caregivers	Burden (did not specify)	Caregiver Burden Scale	Statistically significant differences between both groups regarding total caregivers’ burden and also caregivers’ burden subitems (*P* < 0.05).
		Attitude (did not specify)	Opinions about Mental Illness Scale (OMI)	Statistically significant differences between both groups regarding total OMI and also OMI subitems (*P* < 0.05).

## Discussion

In this review, there was a paucity of local studies to guide the ongoing development of family interventions. Egypt and Jordan are the only two countries from the 22 countries in the Arab world that have published peer-reviewed papers in this area. It is widely acknowledged that there is limited local research to guide the culturally appropriate development of different services in mental health care in the Arab region [50, 51]. The perceived importance of mental health in the Arab world is still low compared to high income countries in the west because health and education budgets are not given enough priority [30]. Okasha et al. [30] stated in their summary regarding health services in the Arab world that some Arab countries are lacking mental health policies. Most countries have less than 30 psychiatric beds per 100,000 of the population. Therefore, the amount and quality of research in this area could be the result of the insufficient resources and services and lack of capability to enhance capacity for conducting high-quality research in this area [30, 52].

### Cultural adaptation of family interventions

The cultural adaptation process of family interventions in the Arab world was consistent with some of the themes reported in previous studies including language, content, concepts and illness models, cultural norms and practice, context and delivery, and family setup [16, 18, 19]. The unique cultural factors that mainly affected content adaptation are the low level of literacy in the Arab world and the unique set of cultural and spiritual values. In the included studies, the language was simplified, and the number of sessions regarding the biological basis of the illness was increased. This is an important cultural consideration because most Arab people relate mental illness to supernatural causes such as black magic and Jinn or being a consequence of God’s punishment [34]. Furthermore, “the evil eye: a powerful jealous look or comment upon the good fortune of another” has a significant impact on the interpretation of mental illness [53]. Consequently, families seek help from traditional healers because they believe that they can treat the spiritual cause by applying religious practices such as exorcism, or miraculous healing [34]. Therefore, some studies strongly recommend incorporating discussion sessions about spiritual factors and cultural-specific belief systems in psychoeducation [22]. However, this may not be sufficient in the Arab world because traditional explanatory models continue to exert a strong influence on help-seeking behaviour. Therefore, researchers should use strategies to incorporate traditional values and beliefs into the intervention [12]. For example, Rami et al. [42] incorporated cultural norms and practices by including folk stories relevant to the cultural and religious beliefs of the participants. These stories are used to resemble the patient’s situation and background and usually include idioms and symbols. Arab folktales are unique in that they are more relatable to everyday life and include a moral lesson [54]. The use of these cultural considerations could help engage families with the biomedical treatment programme. However, the mismatch in explanatory models [55] often persists and can cause patients to drop out at any time.

Exploring a framework that involves a collaboration between traditional healers and mental health professionals may have a positive outcome because the family may feel more comfortable sharing their concerns and accepting the intervention. Many studies of traditional healers encourage such collaboration to improve patient care [56]. In a systematic review by Van der Watt (2018), which includes sixteen articles, it is concluded that participants perceived traditional healers to be effective in treating mental illness, especially when combined with biomedical treatment [57]. This may be the result of meeting the spiritual needs of participants with some religious interventions and offering an explanation for the aetiology of mental disorders.

This review identified two Arabic translated and adapted manuals that can be used in future studies in the Arab world with little modification depending on the specific country’s norms and traditions.

One interesting finding was that all the interventions except Ahmed & Ghaith [44] were in a single-family format. The authors related this to the stigma and discomfort Arabic people feel when discussing the details of their relatives in front of other families. Rami et al. [42] faced this barrier when they piloted the adapted intervention. They found that families preferred individual sessions rather than group sessions. This finding contradicts what was found in the Iranian study [58]. They culturally adapted family psychoeducation group therapy based on needs assessment. Even though Iranian culture is similar to Arab culture, having caregivers in groups increased caregivers comfort in talking to others about their problems. This indicates the necessity of performing needs assessment during the process of cultural adaptation because preferences may differ between various countries in the Arab world.

All papers reported some level of adaptation, with Rami et al. [42] being the most comprehensive. Their process was all-inclusive and included piloting the intervention to examine its acceptability and language simplicity as well as modifying the content accordingly before starting the actual study. This process was congruent with the framework of cultural adaptation proposed by Bernal and Sáez-Santiago [24] to ensure the usefulness and efficacy of delivering the intervention. Another part of the adaptation process was providing the participants with leaflets that contained the primary information taught in each session. This technique ensured reinforced learning and the dissemination of information to other members of the family and the community. These findings were in contrast to those from the systematic review by Chowdhary et al. [16], where they tried to use non-written material to simplify the information. In the Arab world, because of low literacy rates, the use of non-written material such as videos might be more helpful. In contrast, the study by Hasan et al. [43] in Jordan presented psychoeducation in a booklet. The results were promising, and it was reported that it enhanced the participants’ knowledge. Other adaptation themes were not found in Arab world studies. The comparison and contrast of the adaptation process between minority and indigenous populations revealed that adaptation themes such as matching the therapist and clients’ ethnicity as well as other characteristics is more applicable to minority populations in Western countries [59]. In these countries, the process does not require learning about the culture or matching the therapist’s ethnic background with that of the patient. Hence, it can be hypothesised that culturally adapting the intervention for an indigenous population could be a relatively straightforward process if the resources are available compared to adapting it to minority populations in Western countries.

The findings of our review suggest that the adaptation process for family interventions in the Arab world appears robust because it is congruent with the themes from previous systematic reviews. It also gives a clear indication that such interventions are feasible and acceptable enough to be applied in Arab countries.

### Effectiveness of family interventions

Assessing the effectiveness of the interventions was not the main goal of this review. Even if it had been the main goal, the evidence was not available, mainly because of the poor methodology of the included studies. There was clinical heterogeneity in the characteristics of the included studies, such as study design features, methods for diagnosis and evaluation, follow-up, and treatment duration. However, the similarities included recruiting participants from outpatient clinics and using PANSS to measure improvement in clinical symptoms. All studies except Ahmed & Ghaith [44] included both patients and their caregivers in the intervention and used individual instead of group sessions.

The studies showed a positive effect favouring the intervention groups in different outcomes, such as the severity of symptoms, quality of life for service users and their caregivers, social function, adherence to medications, knowledge of schizophrenia, and burden and attitude towards mental illness. The severity of symptoms using the (PANSS) was a common outcome measure used in all studies except Ahmed & Ghaith [44]. Though the studies showed a positive impact on different outcomes, which is in agreement with previous systematic reviews [14, 18, 19], the findings may not have been valid because of the size and poor quality of the included studies. None of the studies, except Hasan et al. [43], had the statistical power to detect a difference in the primary outcome identified. There was substantial heterogeneity in design, types of interventions and outcomes, and only a small number of studies. All these reasons made meta-analysis impossible. Despite this, the meta-analysis by Degnan et al. [19] showed that the different types of interventions had generally similar efficacy, which was due to the common components that are important to specific outcomes; even with different cultures.

The qualitative study by Al-HadiHasan et al. [47] explored the underlying processes for the observed effect of the intervention. Many interesting themes emerged, including “awareness of schizophrenia”, “positive impact on health and wellbeing” and “empowerment and enhanced confidence”. The study clarified the process of applying knowledge regarding schizophrenia in the life of patients and their caregivers. This knowledge improved self-efficacy and empowered participants to take an active role in the treatment plan. It also enabled patients to manage their condition and handle internalised stigma. The intervention helped caregivers to reappraise the demands of better handling of challenging behaviours. Additionally, caregivers were able to control the stressors at home and monitor early signs of relapse. The intervention provided patients and their relatives with knowledge, skills, and coping strategies to manage schizophrenia. This study demonstrated that limited knowledge of mental illness in Arabs was associated with shameful feelings, self-stigma and other negative feelings such as depression, which could lead to non-adherence to treatment and consequently may reduce quality of life. Family members play a significant role in improving the outcome for patients and, consequently, increase their ability to adapt to the role of caregiver. This process can be achieved by educating families to familiarize themselves with patients’ symptoms and behaviours, and equip them with the necessary skills to cope.

### Strengths and limitations

One strength of this review is that Arab participants had a similar culture and language, which could be considered unique compared to previous systematic reviews that included several cultures [14, 19]. Moreover, the searching process was thorough, and the protocol was rigorously followed for study selection, data extraction, analysis and synthesis. The study by Hasan et al. (2017) was followed by a qualitative study to explore the mechanism and process underlying any observed effect. It is well documented that culture has a significant role in how mental illness is interpreted and treated [60]. Therefore, richer data from the qualitative study strengthened the result of the review by providing a deeper understanding of the intervention effectiveness in light of cultural factors for Arabs [61].

A major limitation of this review was the small number of included studies that were variable in design, characteristics and in the components of the interventions. These limitations restricted the conclusion regarding the different objectives of the review, and made the meta-analysis impossible. Furthermore, there was little distinction made between primary and secondary outcomes of the included studies, which caused some confusion and made it difficult to interpret whether the treatment effects differed across outcomes. It is worth noting that the quality of most studies was poor, and these were limited by incomplete information despite efforts to contact authors for clarification. Because the review included only published papers, this could cause publication bias. Therefore, the result should be interpreted with caution.

### Implications

It was not possible, with the available literature, to come to a conclusion regarding the effectiveness of such interventions in the Arab world. However, the recommendation of national and international clinical guidelines to integrate family intervention into routine care, invites more efforts to improve the delivery of such intervention in Arab countries. If this care cannot be integrated fully, at least simple written materials can be offered to patients and their families. The patients and families can then access these materials at their convenience and discuss their understanding with the treating team during follow-up appointments. This may improve the family’s confidence in dealing with the patient’s challenging behaviours and give them clear expectations. This method is simple and requires minimal staff training and resources, which is suitable for use in Arab countries that have limited resources. Arab countries should take action to fight the stigma of mental illness. This could be initiated by conducting mass media awareness campaigns. In addition, when culturally adapting family interventions, researchers may want to collaborate with traditional healers and include culturally relevant discussions about spirituality.

The findings of this review confirmed that the attempts to develop and test culturally adapted family interventions are still quite fragmented in the Arab world. Therefore, a systematic process of developing and evaluating such interventions should be applied for the benefit of a more substantial proportion of the Arab population. Further research using a more suitable methodology, such as an RCT with a large sample and specific outcomes, is recommended to establish and gain a better understanding of the possible effects of such interventions in Arab countries. Another avenue for future research would be to assess family outcomes and the acceptability of such interventions for healthcare professionals and to identify barriers to implementation.

## Conclusion

This study set out to identify the content and characteristics of culturally-adapted family interventions in the Arab world and to determine the strategies used for adaptation. Additionally, it aimed to assess the feasibility, acceptability and effectiveness of these interventions. The present review provides the foundation for future work regarding family interventions in the Arab world and confirms the feasibility of implementing such interventions with some modifications. The data suggested that any alternative family-oriented intervention for schizophrenia - even a short term one - can be better than standard care, and it could improve the outcomes for both patients and their families.

## Supplementary information

**Additional file 1.**

**Additional file 2.**

## Data Availability

The datasets used and analysed during the current study are available from the corresponding author on reasonable request.

## Abbreviations

RCT: Randomised control trial
PROSPERO: Prospective register of systematic reviews
ICD-10: International Classification of Diseases − 10
JBI: The Joanna Briggs Institute
PRISMA: Preferred Reporting Items for Systematic Reviews and Meta-Analyses
PANSS: Positive and Negative Syndrome Scale
