# Supplementary figures and images for: Psychosocial family interventions for relatives of people living with psychotic disorders in the Arab world: systematic review
Source: BMC Psychiatry. 2020 Aug 20;20:413. doi: 10.1186/s12888-020-02816-5 (PMC7441715; doi:10.1186/s12888-020-02816-5)

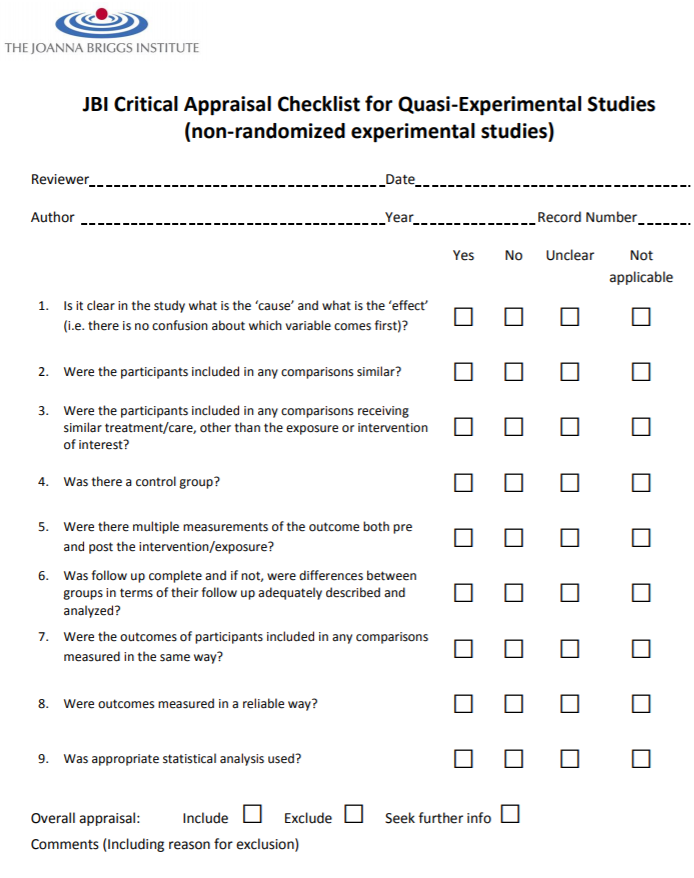

Supplement: Supplementary file 1 — Additional file 1. [file 12888_2020_2816_MOESM1_ESM.docx]

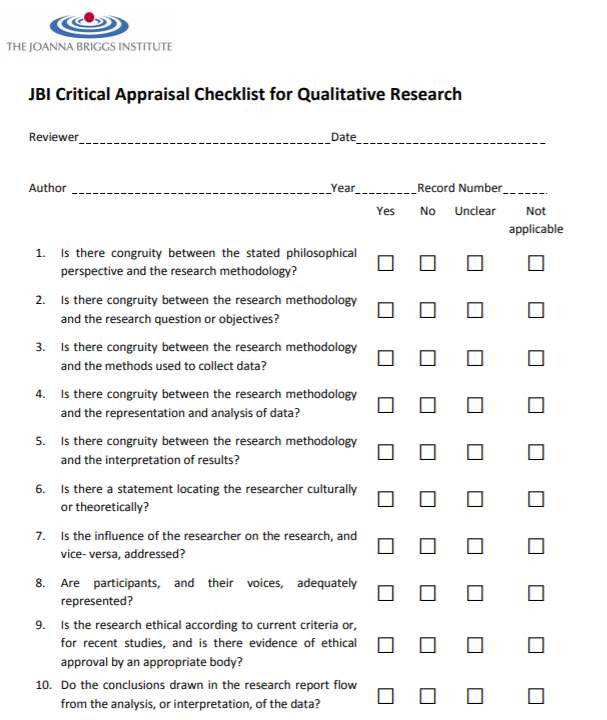

Supplement: Supplementary file 2 — Additional file 2. [file 12888_2020_2816_MOESM2_ESM.docx]
